# Smoking-informed methylation and expression QTLs in human brain and colocalization with smoking-associated genetic loci

**DOI:** 10.1038/s41386-024-01885-4

**Published:** 2024-06-04

**Authors:** Megan Ulmer Carnes, Bryan C. Quach, Linran Zhou, Shizhong Han, Ran Tao, Meisha Mandal, Amy Deep-Soboslay, Jesse A. Marks, Grier P. Page, Brion S. Maher, Andrew E. Jaffe, Hyejung Won, Laura J. Bierut, Thomas M. Hyde, Joel E. Kleinman, Eric O. Johnson, Dana B. Hancock

**Affiliations:** 1 Genomics and Translational Research Center, RTI International, Research Triangle Park, NC USA; 2 Lieber Institute for Brain Development (LIBD), Baltimore, MD USA; 3 Fellow Program, RTI International, Research Triangle Park, NC USA; 4 Department of Psychiatry and Behavioral Sciences, Johns Hopkins University, Baltimore, MD USA; 5 Department of Genetics, University of North Carolina at Chapel Hill, Chapel Hill, NC USA; 6 Department of Psychiatry, Washington University in St. Louis, St. Louis, MO USA; 7 Department of Neurology, Johns Hopkins University, Baltimore, MD USA

**Keywords:** Gene expression, DNA methylation, Genetic interaction

## Abstract

Smoking is a leading cause of preventable morbidity and mortality. Smoking is heritable, and genome-wide association studies (GWASs) of smoking behaviors have identified hundreds of significant loci. Most GWAS-identified variants are noncoding with unknown neurobiological effects. We used genome-wide genotype, DNA methylation, and RNA sequencing data in postmortem human nucleus accumbens (NAc) to identify *cis*-methylation/expression quantitative trait loci (meQTLs/eQTLs), investigate variant-by-cigarette smoking interactions across the genome, and overlay QTL evidence at smoking GWAS-identified loci to evaluate their regulatory potential. Active smokers (*N* = 52) and nonsmokers (*N* = 171) were defined based on cotinine biomarker levels and next-of-kin reporting. We simultaneously tested variant and variant-by-smoking interaction effects on methylation and expression, separately, adjusting for biological and technical covariates and correcting for multiple testing using a two-stage procedure. We found >2 million significant meQTL variants (*p*_adj _< 0.05) corresponding to 41,695 unique CpGs. Results were largely driven by main effects, and five meQTLs, mapping to *NUDT12*, *FAM53B*, *RNF39*, and *ADRA1B*, showed a significant interaction with smoking. We found 57,683 significant eQTL variants for 958 unique eGenes (*p*_adj _< 0.05) and no smoking interactions. Colocalization analyses identified loci with smoking-associated GWAS variants that overlapped meQTLs/eQTLs, suggesting that these heritable factors may influence smoking behaviors through functional effects on methylation/expression. One locus containing *MUSTN1* and *ITIH4* colocalized across all data types (GWAS, meQTL, and eQTL). In this first genome-wide meQTL map in the human NAc, the enriched overlap with smoking GWAS-identified genetic loci provides evidence that gene regulation in the brain helps explain the neurobiology of smoking behaviors.

## Introduction

Genetic variants that act as quantitative trait loci (QTLs) for gene regulatory features, such as DNA methylation (DNAm) and RNA expression (RNAexp) levels, are pervasive across the genome [1–5] and are enriched among disease-associated loci [6–8]. Genome-wide variants have been characterized for their QTL effects across different regulatory features and tissues by the Genotype-Tissue Expression (GTEx) project [1, 9] and others [2, 4]. However, understanding of tissue-specific QTL effects in the context of exogenous exposures remains limited. This is an important next step to better understand mechanisms underlying complex disease processes.

Cigarette smoking is an exogenous exposure associated with altered gene regulation, as shown in both blood [10] and brain [11, 12]. Although some gene regulatory features are shared across tissues, others can be highly tissue-specific, with brain tissues showing the most distinct profiles compared with other tissues [13]. Notably, poor correlations have been observed between RNAexp [1] and DNAm levels [14], and differences in the QTLs implicated [15] in brain versus other tissues means that more accessible tissues, such as blood, provide insufficient proxies for brain-specific regulatory mechanisms. Even within the brain, gene regulation and related QTLs can differ by tissue [1]. It is therefore critical to focus on disease-relevant tissues in the brain by relying on human postmortem tissues to assess the neurobiological underpinnings of disease-associated variants and their target genes and the neurobiological impact of cigarette smoking. This study aims to shed light on the intricate mechanisms linking genetically driven gene regulation in the context of cigarette smoking in the brain, offering crucial insights into the broader health implications.

Postmortem human brain studies of various addiction-relevant tissues helped explain earlier genetic loci identified via genome-wide association study (GWAS) analyses for cigarette smoking behaviors, specifically *cis*-expression QTL (*cis*-eQTL) and *cis*-methylation QTL (*cis*-meQTL) variants [16–18]. Since then, the genetic architecture of smoking has rapidly evolved, with GWAS sample sizes exceeding 3 million in the GWAS and Sequencing Consortium of Alcohol and Nicotine use (GSCAN), resulting in hundreds of genome-wide significant loci identified for smoking initiation, age at initiation, cigarettes per day, and cessation [19, 20]. The underlying neurobiology is unknown for most of these loci, underscoring the need to investigate their functional effects that may drive smoking behaviors.

Multiple brain regions are involved in cigarette smoking, and addiction more broadly, as part of a three-stage cycle [21]. In this context, we specifically focus on nucleus accumbens (NAc), a core tissue of the first stage of the addiction cycle (binge/intoxication), given its integral role in cognitive processing of motivation, pleasure, reward, and reinforcement [22]. We present a comprehensive study that maps genome-wide *cis*-eQTL and *cis*-meQTL variants and investigates variant × cigarette smoking interactions in the NAc of decedents from the Lieber Institute for Brain Development (LIBD). To our knowledge, this resource provides the first genome-wide meQTL map in human NAc, and the co-occurring *cis*-meQTL/eQTL maps in the same decedents enable a deeper understanding of shared causal mechanisms. Our study uniquely leverages smoking status postmortem to discern variants whose quantitative effects may vary upon smoking exposure, in contrast to previous phenotype-agnostic QTL maps in the human brain [1, 15].

## Methods

### Human postmortem NAc samples

Postmortem human NAc tissues were obtained from the LIBD brain collection at autopsy, as previously described [11, 23]. Exclusion criteria included DSM-5 psychiatric or substance use disorders other than nicotine, and others (Supplementary Materials). Information regarding demographics, substance use history, and current smoking status was collected from a 36-item telephone questionnaire administered to the next-of-kin. Cotinine biomarker measures from brain and/or blood samples were measured using a standard toxicology screen (National Medical Services Labs, Inc., Willow Grove, Pennsylvania). As in Markunas et al., smoking cases were defined by cotinine levels above 12 ng/mL in blood and 12 ng/g in the brain, a threshold that differentiates between active and passive smoking [24]; and by a next-of-kin report of current smoking [11]. Controls (nonsmokers) were defined by cotinine levels below 12 ng/mL (blood) or 12 ng/g (brain) and a next-of-kin report of no current smoking.

### Genotype data

The genotype data used in this study were obtained from a superset of samples that were genotyped and imputed as part of the full LIBD cohort, using previously described procedures [23]. Briefly, samples from the full cohort were genotyped across multiple Illumina microarray platforms (see Supplementary Methods) and underwent a standard quality control (QC) protocol [25] to remove low-quality and low-frequency variants. Haplotypes were phased using SHAPEIT [26] and then imputed using IMPUTE2 [27] with reference to 1000 Genomes phase 3. Imputed genotype dosages were converted to hard-call genotypes for variants with imputation posterior probabilities >0.9.

### DNAm and RNA-seq data generation and processing

DNA and RNA were extracted from NAc samples of 239 eligible LIBD decedents, as described previously [28, 29]. DNAm was measured using an Illumina Human MethylationEPIC BeadChip. As described before [11], DNAm data processing and QC were conducted using the R package *minfi* [30]. DNAm β-values were calculated and used in the meQTL analyses, representing the percentage of DNAm at each CpG (ratio of methylated intensities relative to the total intensity). See Supplementary Materials for more details.

In addition, the DNAm processing, sample and probe exclusions, and modeling information are described extensively in the Supplementary Materials of Markunas et al. [11].

Following RNA extraction, samples were sequenced using paired-end 100 bp reads on an Illumina HiSeq3000 at LIBD [11, 31]. RNA-seq samples were pseudomapped using *Salmon v1.1.0* [32]. Transcript quantifications were aggregated to the gene-level counts using *tximport v1.12.3* [33], resulting in 60,240 GENCODE v34 genes. Samples were excluded based on RNA-seq quality metrics, low RNA integrity (RIN) score, discrepancies between self-reported sex and chromosome *Y* gene expression, discrepancies between RNA and DNA derived genotype data, and missing genotype data. Sample-level QC removed 36 samples, resulting in a post-QC RNA-seq sample size of 203. Lowly expressed genes were then removed using the exclusion criteria of ≥90% of samples with ≤10 gene counts or ≤1 transcripts per million value. For eQTL mapping, GRCh37 human genome reference coordinates were used for all gene annotations to align with the genotype data genome build. In total, 16,274 genes were considered for eQTL mapping. See Supplementary Materials for more details.

### Methylation and expression quantitative trait loci (meQTL/eQTL) mapping

We performed *cis*-meQTL mapping using imputed genetic variants with an MAF > 0.05 and DNAm intensity β-values of probes proximal, within 500 kilobases (kb) up or downstream, to these variants. Four different meQTL mapping regression models were fit: (1) a “baseline” model to test for association between genetic variants and DNAm β-values across both smoking cases and controls; this model included age at death, sex, estimated non-neuronal cell-type proportion [34], principal component 1 (PC1) for DNAm array negative control probes, PC1 for imputed genotypes, and PC2 for imputed genotypes as covariates (see Supplementary Methods for model selection details); (2) a smoking cases–only model similar to the baseline model; (3) a smoking controls–only model similar to the baseline model; and (4) an interaction model to test for associations of a genetic variant-by-smoking status interaction with DNAm β-values. All models used rank-inverse normal transformed (RINT) DNAm β-values. The smoking cases–only and controls–only models are needed to generate summary statistics used to conduct stratified two degrees-of-freedom (2DF) tests. The stratified 2DF test jointly tests for genetic variant main effects and genetic variant-by-smoking status interaction effects [35]. Additional details are provided in the Supplementary Methods.

A similar framework was applied for *cis-*eQTLs. Baseline, smoking cases–only, smoking controls–only, and interaction eQTL models were fit for each imputed genetic variant and NAc expression levels of genes proximal (within 500 kb of gene body) to these variants. Counts for a given gene underwent median-of-ratios normalization [36] and RINT to obtain values used to represent the gene’s expression level. The covariates included in each model were age at death, sex, exon mapping rate, ribosomal RNA mapping rate, PC1 for imputed genotypes, PC2 for imputed genotypes, and four latent variables estimated by PEER v1.3 [37] to account for additional unmeasured sources of confounding (see Supplementary Methods).

To account for multiple hypothesis testing in the meQTL/eQTL models and stratified 2DF tests, *p* values were corrected using a conversative two-stage approach to mitigate inflation associated with single-stage approaches when applied to QTL mapping [38]. This hierarchical approach first accounts for association tests across variants for a given DNAm probe/gene, then accounts for tests across all probes/genes. In the first stage, all nominal *p* values for a given probe/gene were adjusted using EigenMT [39]. In the second stage, EigenMT adjusted *p* values were further corrected by the number of probes/genes tested. Any initial two-stage adjusted *p* value smaller than a Bonferroni corrected *p* value was assigned the latter *p* value as the final two-stage adjusted *p* value. This ensures that the two-stage adjustment stringency does not exceed the family-wise error rate control of Bonferroni correction. A two-stage adjusted *p* value cutoff of 0.05 was used to identify statistically significant QTLs.

### Gene Set Enrichment Analysis (GSEA) for eGenes and meQTL CpGs

We used the GSEAPreranked tool from GSEA v4.3.2 [40] to test for enrichment of smoking-associated eGenes and meQTL CpGs in the MSigDB v2023.1.Hs gene set collections *C2 canonical pathways* and *C5 Gene Ontology gene sets*. Only gene sets with 15–500 genes were included. CpGs were assigned to genes using the Infinium MethylationEPIC v1.0 B5 Manifest, resulting in 11,772 CpGs from the interaction meQTL mapping model with at least one assigned gene. Each gene was ranked using the largest −log_10_-transformed, smoking interaction *p* value for a given gene from QTL mapping. Weighted Kolmogorov–Smirnov–like statistics were computed for the enrichment scores, and *p* values were determined using 1000 gene set permutations. Significant pathways were selected based on false discovery rate (FDR) < 0.10, which was selected to maximize biological discovery and yet be more stringent than the default threshold of 0.25.

### QTL enrichment testing for GSCAN-identified genetic variants

We conducted variant-based enrichment testing to assess whether genome-wide significant GSCAN loci were enriched for meQTLs or eQTLs. We obtained GSCAN summary statistics from the University of Minnesota’s Data Repository for U of M (10.13020/3b1n-ff32), focusing on GSCAN’s GWAS results from 2019 to capture genetic loci with variants that commonly occur and have the largest effect sizes on smoking: *N* up to 1.2 million individuals, depending on the smoking trait analyzed [19]. For meQTL enrichment analysis, we compared the *p* value distributions from stratified 2DF tests (i.e., stratified 2DF meQTL mapping) between GSCAN variants and a set of randomly matched variants. The random matched variant set was designed to be 10 times the size of the GSCAN variant set. The GSCAN variant set included linkage disequilibrium (LD)-pruned, genome-wide significant variants reported by GSCAN, across all four smoking traits, that were also available in our meQTL mapping (361 variants, each representing an independent significant locus). The matched variant set included 3,600 LD-pruned variants selected using SNPsnap [41]. For variants with multiple stratified 2DF *p* values (i.e., proximal to multiple CpG sites), only the smallest *p* value was retained. The meQTL stratified 2DF *p* value distributions for the GSCAN and matched variant sets were tested for equality using a two-sided Kolmogorov–Smirnov test. The eQTL enrichment analysis followed the same procedure as the meQTL enrichment analysis. The GSCAN variant set included 305 variants because the overlap with variants from eQTL mapping differed from meQTL mapping. The SNPsnap-constructed, matched variant set included 3050 (305 × 10) variants.

### Colocalization between GSCAN smoking GWAS and meQTL/eQTL mappings

We tested whether meQTL or eQTL signals from the baseline model QTL mapping colocalized with GSCAN loci for smoking initiation, age at initiation, cigarettes per day, and cessation using the *coloc v5.1.0* R package. We describe this analysis for meQTLs, but an equivalent framework was applied for eQTLs. All DNAm probes with an meQTL mapping *cis*-window that overlapped with a GSCAN locus were considered for colocalization. For a given probe, the colocalization test region spanned all genetic variants that were (1) included in the meQTL mapping for the probe and (2) tested in the GSCAN GWAS (Fig. S1). Summary statistics from GSCAN and the baseline model meQTL mapping were used. Significant colocalization required a *coloc* posterior probability >0.8 for hypothesis 4 (both traits are associated and share a single causal variant) and a two-stage, adjusted *p* < 0.05 in the baseline meQTL analysis (Supplementary Materials).

For GSCAN loci that showed evidence of colocalization, HyPrColoc was applied to assess whether these colocalizations resulted from the same region of the locus [42]. Each HyPrColoc test included only one GSCAN trait, eGene, and CpG. For each HyPrColoc test, a genetic variant was included as input only if it had summary statistics available from the baseline model me/eQTL mapping and GSCAN GWAS for the smoking trait. Significant colocalization was defined as a GSCAN trait–eGene–CpG triplet having posterior probability >0.8.

## Results

### Overview

Of the available 239 decedents, 201 RNA-seq samples and 220 DNAm samples, with genotype and smoking data, remained following QC, including 198 samples in both datasets (intersection) and 223 samples (52 cases and 171 controls) with either RNA-seq or DNAm data (union) (Table 1). Of the cases, 50% had African ancestry (AA) and 50% had European ancestry (EA) based on next-of-kin report and genotype confirmation. The manner of death differed slightly among cases and controls, but age, sex, and postmortem interval were similar (Table 1).

**Table 1:** Characteristics of decedents included for methylation and expression quantitative trait loci mapping in nucleus accumbens.

	Case (*N* = 52)	Control (*N* = 171)	*p* value
Age, years			
Mean (SD)	47.4 (12.5)	45.6 (14.1)	0.37
Median [Min, Max]	47.2 [18.8, 73.9]	48.8 [17.4, 71.8]	
Sex			
Female	14 (26.9%)	44 (25.7%)	0.86
Male	38 (73.1%)	127 (74.3%)	
Race			
African Ancestry	26 (50.0%)	78 (45.6%)	0.60
European Ancestry	26 (50.0%)	93 (54.4%)	
Manner of Death			
Accident	2 (3.8%)	29 (17.0%)	0.04
Homicide	3 (5.8%)	13 (7.6%)	
Natural	46 (88.5%)	128 (74.9%)	
Unknown	1 (1.9%)	1 (0.6%)	
Postmortem Interval, hours			
Mean (SD)	32.9 (15.9)	29.1 (12.2)	0.12
Median [Min, Max]	31.0 [0, 68.0]	26.0 [9.00, 67.5]	

*p* value calculated with Fisher’s exact test for categorical variables and a *t* test for quantitative variables.

**Fig. 1:**
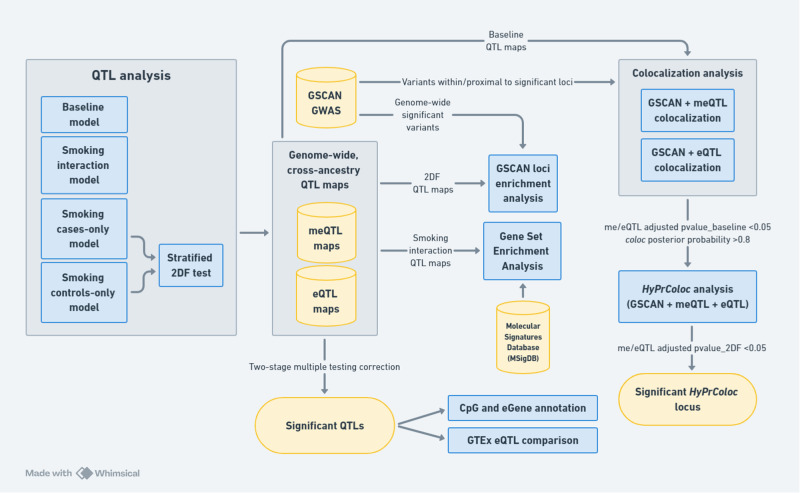
Analysis workflow. QTL analyses were conducted using DNA methylation, gene expression, and genotype data and three different QTL models to produce genome-wide *cis*-eQTL and *cis*-meQTL maps for nucleus accumbens. Two-stage multiple testing correction was applied to these QTL maps to identify significant QTLs that underwent further annotation. QTL maps were also integrated with GSCAN GWAS summary statistics to perform genetic variant enrichment testing and colocalization analyses. Smoking interaction QTL maps were used in conjunction with the Molecular Signatures Database to conduct gene set enrichment analysis and identify biological pathways enriched for CpGs/genes associated with smoking status and genotype.

We generated single data-type QTL maps. Then each QTL type independently underwent colocalization analysis with GSCAN GWAS summary statistics, and joint colocalization analyses across data types were performed for significant me/eQTLs (Fig. 1).

### Genome-wide *cis*-meQTL maps

DNAm in NAc and genotype data were available for 52 smoking cases (26 EA, 26 AA) and 168 smoking controls (75 EA, 93 AA). In all, 11,206,899 variants were used in the initial analysis and 784,843 CpGs, resulting in 1,748,985,510 meQTL tests. After applying two-stage multiple testing correction, we identified 2,552,641 significant meQTL variants targeting 51,315 unique CpGs (Table S1) -which is restricted to the lead variant due to size; full results are available at synapse.org, 10.7303/syn50996324).

To identify the most robust signals, we performed post hoc filtering, keeping only meQTLs where the top variant had a minor allele frequency (MAF) ≥ 0.05 and missingness ≤0.10 in both ancestries, leaving 41,695 unique CpGs. The top five most significant meQTLs include CpGs that map to *PITRM1*, *KDM3B*, *ARID1B*, or *MTL5* (Table S1).

### Genome-wide *cis*-eQTL maps

Gene expression (RNA-seq) in NAc and genotype data were available for 47 smoking cases (24 EA, 23 AA) and 156 smoking controls (72 EA, 84 AA). These data were previously used to map eQTLs agnostic to the smoking phenotype [5]. In the present study accounting for variant-by-smoking interaction, we identified 83,095 significant eQTL variants from 1050 eGenes after multiple testing correction. Of these, 57,683 eQTL variants targeting 958 unique eGenes remained after post hoc filtering was applied to identify the most robust signals, keeping only eQTLs where the top variant had an MAF ≥ 0.05 and missingness ≤0.10 in both ancestries (Table S2). All significant results are shown in Table S2. Table S3 is filtered to the lead variant for each significant eGene (*N* = 1050). The 10 most significant eGenes were *RPL9*, *ZSWIM7*, *GATD3B*, *RPS28*, *XRRA1*, *TMEM161B-AS1*, *CUTALP*, *NIPBL-DT*, *ZNF718*, and *SPATA7* (Table S3). Full results are available at synapse.org (10.7303/syn50996324).

**Fig. 2:**
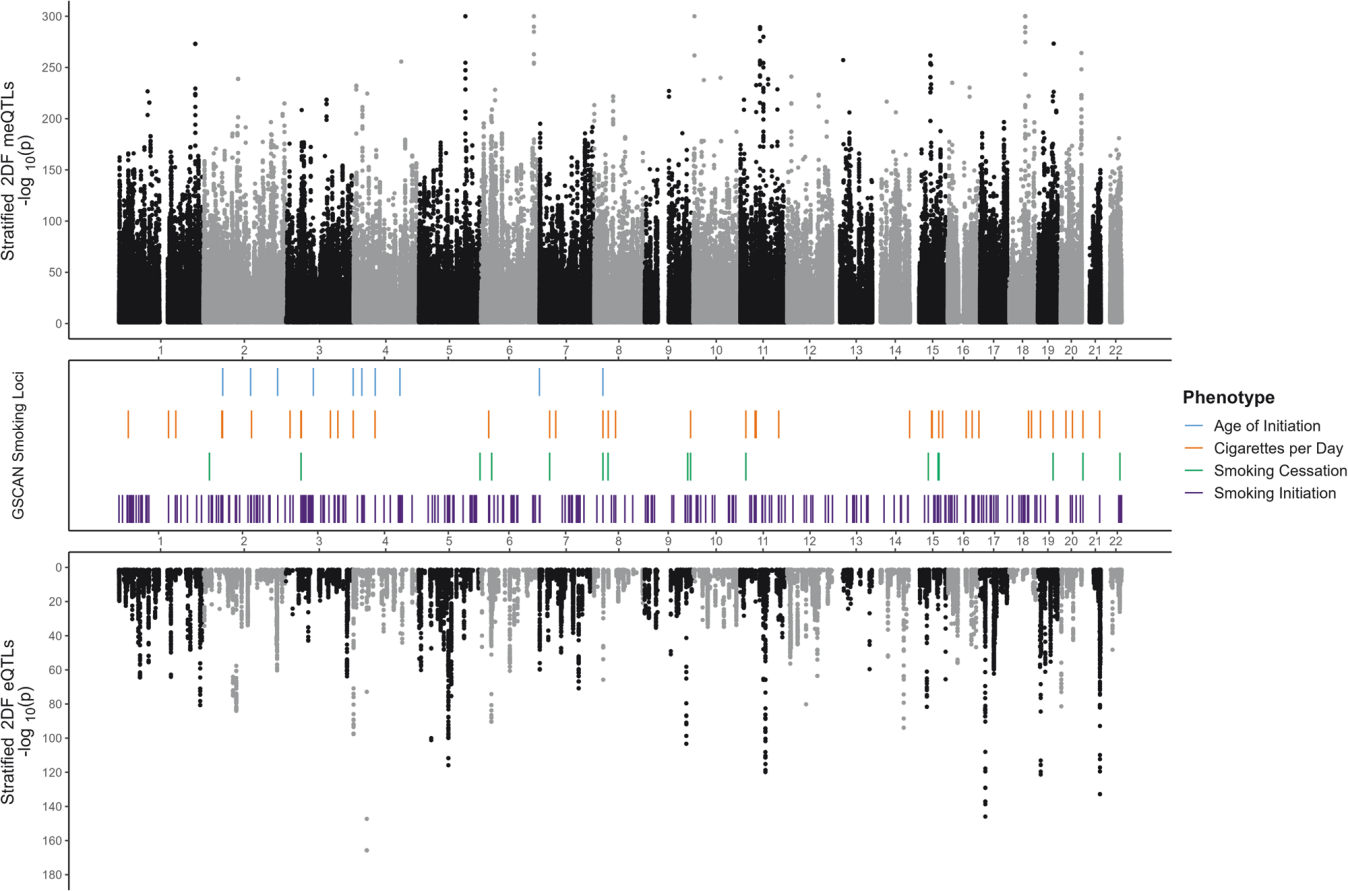
Miami plot for stratified 2DF meQTL and eQTL maps. Stratified 2DF QTL mapping −log_10_ nominal *p* values for significant (2DF adjusted *p* ≤ 0.05) genetic variant–CpG probe (top panel) and genetic variant-gene associations tests (bottom panel) are displayed as a function of genome position (*x*-axis). Genomic locations of significant GSCAN GWAS loci for four smoking phenotypes are denoted by the bars in the middle panel.

Both meQTLs and eQTLs were pervasive throughout the genome (Fig. 2). When ﻿we compared the eQTL to meQTL results, 562/1050 (54%) significant eGenes overlapped genes annotated to significant meQTL CpGs. Additionally, data from 655 (62%) robust eGenes (after MAF and missingness filtering) identified in our analysis were available in the NAc GTEx data. Of these 655, 509 (78%) met a Bonferroni correction significance threshold (0.05/655 = 7.6e-5) (Table S3, GTEx column: *Nominal* p-value). Thus, we observed a high correlation when comparing NAc eQTL results between our study and GTEx.

### Smoking interaction effects with meQTLs/eQTLs

To identify meQTLs/eQTLs that differed by smoking, we compared the 2DF QTL test results with those of the baseline models and interaction models. The meQTLs were primarily driven by main effects, with few showing evidence for interaction. Of the 41,695 significant unique CpGs, only five demonstrated strong evidence of a smoking interaction (Table 2, Fig. S2) based on Bonferroni correction (significance threshold of 0.05/41,695 = 1.2e-6). No eQTLs showed evidence of an interaction (significance threshold of 0.05/877 unique eGenes=5.7e-5) after filtering by MAF and missingness.

**Table 2:** Five meQTLs with evidence of significant variant × smoking interactions.

Variant	CpG	CpG distance from top variant (bp)	Gene	*p*_2DF Adjusted_	*p*_main effect_	*p*_interaction_
rs16965753	cg05215806	87468	*—*	0.005	0.76	2.5e-07
rs73190041	cg06105925	43102	*NUDT12*	0.002	1.3e-06	1.5e-07
rs4962681	cg17990900	5083	*FAM53B*	1.02e-08	2.9e-06	5.8e-07
rs7770811	cg20119745	78880	*RNF39*	0.045	2.7e-05	6.4e-07
rs6884129	cg25315648	104	*ADRA1B*	3.41e-20	1.36e-17	8.7e-07

See Table S1 for full annotations.

Because use of stringent significance thresholds may miss subtle smoking interactions with individual variants, we performed GSEA using genes ranked based on QTL–smoking interaction* p* values to identify QTL-enriched biological processes and pathways that may be altered by smoking. For meQTL-smoking interactions, five pathways related to the synaptic cleft (neurotransmission site between pre- and postsynaptic membranes) were implicated. For eQTL-smoking interactions, cell cycle and wound response processes (cellular changes resulting from a stimulus indicated damage to an organism) were enriched (Table S4).

### GWAS-identified variants that exert QTL effects on their target genes

To test the overlapping evidence of GWAS-identified variants as QTLs, we performed an enrichment analysis using a two-sample Kolmogorov–Smirnov test that compared NAc meQTL and eQTL *p* value distributions at GSCAN significant variants with a random set of variants. We found that GSCAN variants were significantly enriched for meQTLs (*p* = 0.005) but not for eQTLs (*p* = 0.3). This aligns with our observation that more meQTLs than eQTLs overlap GSCAN variants (Tables S5–S7). For comparison, using the same test with NAc eQTLs from GTEx, we found an enrichment *p* = 0.07.

### Smoking meQTL, eQTL, and GWAS colocalization

We performed colocalization analyses of GSCAN’s GWAS results with our QTL maps to characterize heritable components of smoking that exert QTL effects. First, we performed pair-wise analyses (meQTL + GWAS, eQTL + GWAS), starting with GSCAN significant loci. Because a single locus may include more than one unique CpG or eGene, many colocalization analyses were performed per region. In general, we observed more colocalization of meQTLs than eQTLs with the GWAS loci (Tables S8–S10). Four genome-wide significant CpGs colocalized across two phenotypes (cigarettes per day and age of initiation): cg12293539 at *MAML3* and cg00622170, cg11254171, and cg18236429 at the *NOP14/NOP14-AS1* locus.

**Fig. 3:**
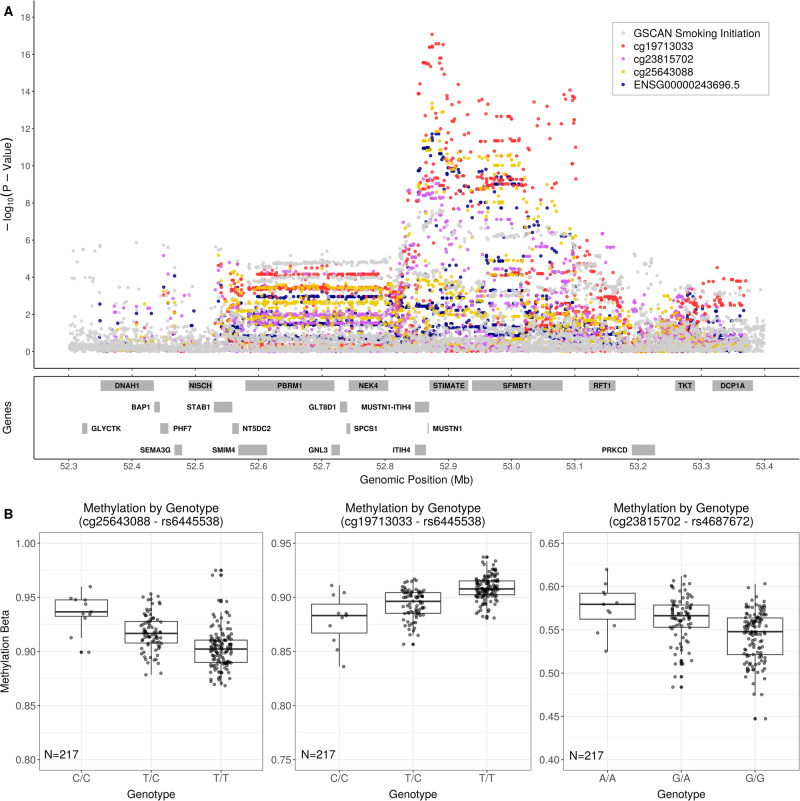
GSCAN smoking initiation GWAS, meQTL, and eQTL association test −log_10_*p* values near GSCAN locus chr3: 52386605–54266212. **A** −log_10_ nominal *p* values for associations between genetic variants and smoking initiation, expression of three CpGs, and gene expression for the eGene identified by HyPrColoc with colocalization probability >0.8. Only genetic variants that overlapped across summary statistics for GSCAN, meQTL mapping, and eQTL mapping are plotted. Locations of genes included in the eQTL mapping are annotated in the bottom of the panel. **B** Genotype-by-CpG plots for the CpGs in Fig. 3A showing CpG probe intensity levels partitioned by genotype.

Next, we performed colocalization using a method (HyPrColoc) that can incorporate meQTLs, eQTLs, and smoking GWAS in a single analysis. Focusing on GSCAN GWAS loci that colocalized with both a significant eQTL and meQTL, we confirmed the GWAS–eQTL–meQTL colocalizing region at the GSCAN smoking initiation locus chr3: 52386605–54266212. Three variant–CpG–eGene combinations had significant colocalization, all involving ENSG00000243696 (predicted read-through of *MUSTN1-ITIH4*) as the eGene with (1) rs6445538 and cg25643088, (2) rs6445538 and cg19713033, and (3) rs4687672 and cg23815702 (Fig. 3A). The C allele of rs6445538 and the A allele of rs4687672 are associated with increased smoking initiation with very modest effect sizes: 0.023 for both [19, 43]. These alleles are associated with increased ENSG00000243696 gene expression (Table S2: Column: β_baseline_), increased DNA methylation at cg25643088 and cg23815702, and decreased DNA methylation at cg19713033 (Fig. 3B, Table S1: Column: β_baseline_).

## Discussion

Cigarette smoking remains highly prevalent and a leading cause of death globally, despite decades of research into the health consequences and public health campaigns to curb smoking [44, 45]. Addiction to cigarette smoking is a complex, multi-stage process involving a neuronal rewards system that includes the NAc region of the brain [21, 46]. The NAc is known to have a role in cognitive processing of motivation, reward, and reinforcement, which are essential to the first stage of addiction (binge/intoxication) [22]. This study employed methods to better understand the functional effects of heritable factors that influence smoking behaviors.

QTLs are pervasive throughout the genome and provide valuable insight into tissue-specific gene regulation. The GTEx project, widely used for exploring eQTLs, recently released a large-scale meQTL dataset encompassing nine human tissues [47]. However, this dataset did not capture brain tissues. To our knowledge, ours is the first genome-wide meQTL map in human NAc and is shared publicly as a new resource for the scientific community.

We found few significant variant-by-smoking interaction effects and conclude that most QTLs in the NAc may not differ by smoking. While some interactions with small effect sizes may exist, they may require larger sample sizes to detect. We investigated this possibility using a pathway analysis ranked by evidence of smoking interactions, and we identified pathways related to cellular damage, cell cycle, and the synaptic cleft, where the nicotinic acetylcholine receptors play an important role in regulating neurotransmission [48]. Five individual meQTLs differed significantly between smokers and nonsmokers, including Nudix Hydrolase 12 (*NUDT12*), Family with sequence similarity 53 member B (*FAM53B*), and Ring Finger Protein 39 (*RNF39*). *NUDT12* plays a role in nicotinate and nicotinamide metabolism [49]. Interestingly, *NUDT12* was identified in a transcriptome analysis of neurons following chronic nicotine exposure [50] and lies within a QTL interval for nicotine sensitivity in mouse studies [51, 52]. *FAM53B* has been associated with cocaine dependence [53]. Differential DNAm at *FAM53B* has been observed in COPD cases compared with controls [54]. *RNF39* was found to be differentially methylated in a study of marijuana use [55] and other smoking-related DNAm changes [56, 57]. Together, these represent biologically plausible genes whereby smoking may alter genetically driven gene regulation in NAc.

We employed colocalization analyses [5] to identify trait-associated variants that act as regulators of DNAm or gene expression. We generally observed more overlap between the GWAS-associated variants and meQTLs than eQTLs.The weaker overlap of GWAS variants and eQTLs has been noted elsewhere [58], and this pattern was also supported by our enrichment analyses, and may relate to the magnitude of meQTL tests. However, the same pattern held when we looked only at QTLs that survived genome-wide multiple testing correction, whereby declaring meQTLs as statistically significant was based on a more stringent threshold than eQTLs and is consistent with studies of the prefrontal cortex [59], blood [60], and other tissues [47].

One genomic region showed robust evidence of colocalization with all three data types, highlighting novel functional evidence where changes to DNAm and gene expression may help explain the neurobiology underlying a smoking initiation associated heritable factor. The primary gene indicated is a predicted read-through of the Musculoskeletal, Embryonic Nuclear Protein 1 (*MUSTN1*) and inter-alpha-trypsin inhibitor, heavy chain 4 (*ITIH4*) genes. Little is known about the read-through transcript. *MUSTN1* is known to have a role in skeletal muscle homeostasis, chondrocyte differentiation, and limb morphogenesis [61]. Schizophrenia-associated variants in *ITIH4* have been shown to regulate expression of *ITIH4* in the prefrontal cortex [62], and variants in *ITIH4* is a biomarker for COPD [63, 64]. *MUSTN1* and *ITIH4* are expressed at low levels in the brain and moderately expressed in skeletal muscle [1, 65, 66]. There is evidence that changes to skeletal muscle homeostasis can influence the physiology of the brain [67, 68]. However, we did not detect a significant eQTL for *MUSTN1* or *ITIH4* in our eQTL analysis, indicating that the *MUSTN1-ITIH4* read-through may warrant further functional interrogation to decipher how its role may differ from *MUSTN1* and *ITIH4*.

This study has limitations to consider in interpreting the findings. First, because this study utilized an understudied brain tissue collected from decedents with a unique set of multi-omics data types and smoking status, sample availability was limited to 52 smoking cases and 171 smoking controls. This constrained sample size may have limited our ability to identify interaction effects, and it limited interrogation of ancestry- or sex-specific effects and extension into independent replication datasets. Also, QTL mapping results in millions of tests, increasing the chance of type I error. We accounted for multiple testing using a two-stage correction strategy designed for this type of study [39]; however, the possibility of type I error remains. Furthermore, despite the concordance between eGenes identified from eQTL mapping and those reported by GTEx, the two-stage correction strategy we applied has reduced sensitivity to detect eQTL relative to the single-stage FDR correction approach applied by GTEx, resulting in a lower number of reported eGenes in our study despite comparable sample size.

Finally, the QTL–GWAS colocalization was based on the first GSCAN meta-analysis, including up to 1.2 million individuals [19]. GSCAN recently released an updated meta-analysis with >3 million individuals [20]. Although comparison with the updated GSCAN meta-analysis might result in the identification of additional QTL–GWAS colocalization signals, given the sample size available with multi-omics data in our postmortem human brain study, we would have less statistical power to detect colocalization with the lower MAF variants in GSCAN2, and these variants are not well captured by our imputed genotype data. The present study focused on genetic loci with common variants with the largest effect sizes on smoking.

This study had several strengths. It is the first to provide a genome-wide meQTL map in human NAc, a relatively understudied brain tissue with an important role in the addiction cycle. Case definitions were carefully established based on corroborating evidence from several sources, including blood- and brain-based toxicology screens with confirmation by next-of-kin reports. Therefore, misclassification is unlikely. We used the 2DF test, which achieves power similar to that of a standard 1DF test when no interaction is present and simultaneously improves power when interaction effects are present [35]. We used conservative thresholds to identify the most robust signals, and the full results are provided for researchers to explore additional signals. To our knowledge, the present study represents the first large-scale formal testing of colocalization for GSCAN-identified loci with QTLs in human brain, identifying novel target genes and providing insight into the neurobiological function of smoking-associated heritable factors in relation to both DNAm and gene expression.

Overall, this multi-ancestry, multi-omics study of decedents with smoking status known and accounted for provides a unique resource for interrogating regions across the genome for their influence on gene regulation, cigarette smoking behaviors, and other complex conditions involving the NAc. Future studies may use these data to compare QTLs across other brain tissues to gain insights into tissue-specific regulation and to further investigate the neurobiology underlying other disease processes.

## Supplementary information

Supplementary Methods

Supplementary Table S1

Supplementary Table S2

Supplemental Table S3

Supplementary Table S4

Supplementary Table S5

Supplementary Table S6

Supplementary Table S7

Supplementary Table S8

Supplementary Table S9

Supplementary Table S10
